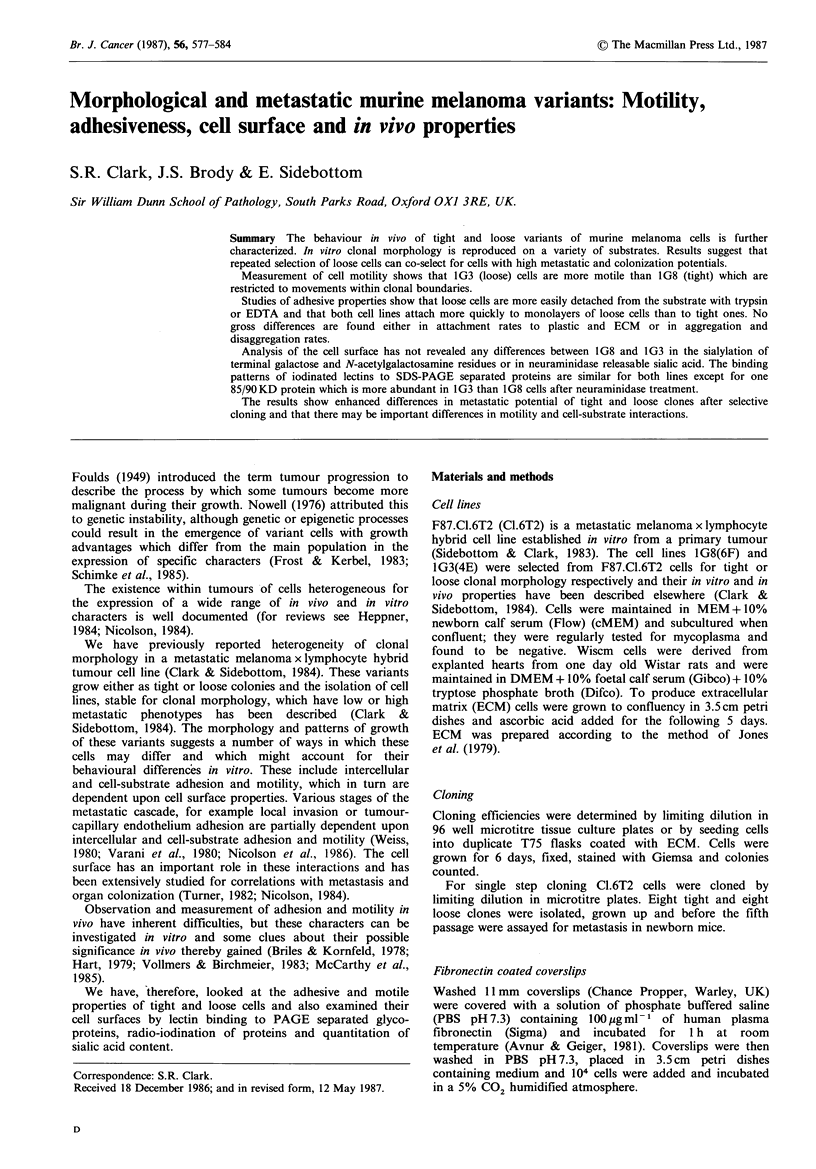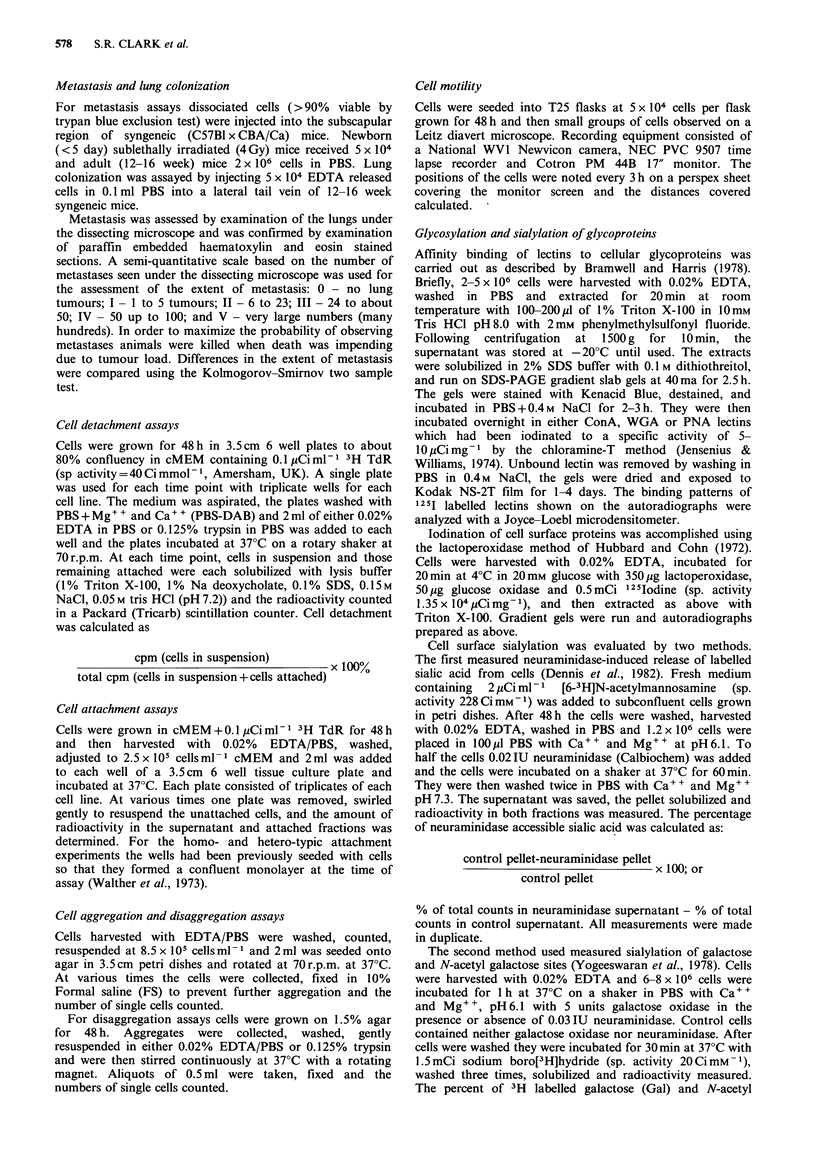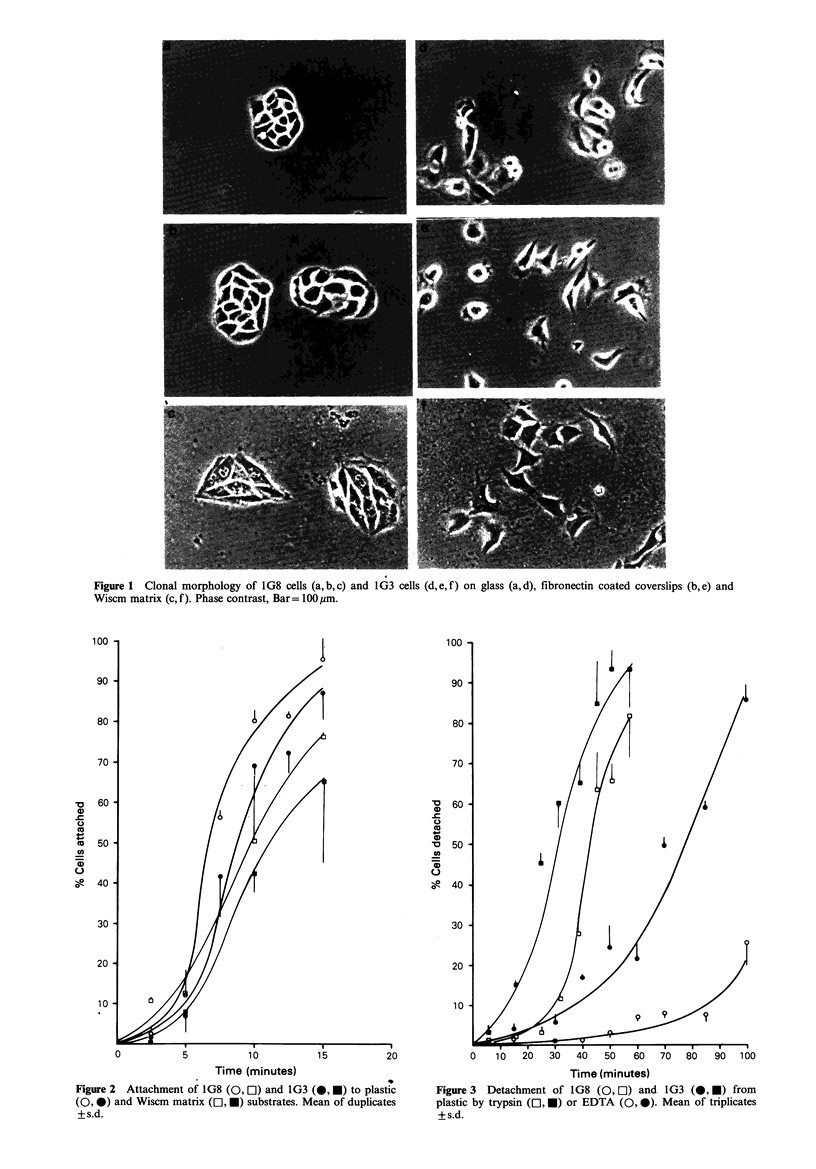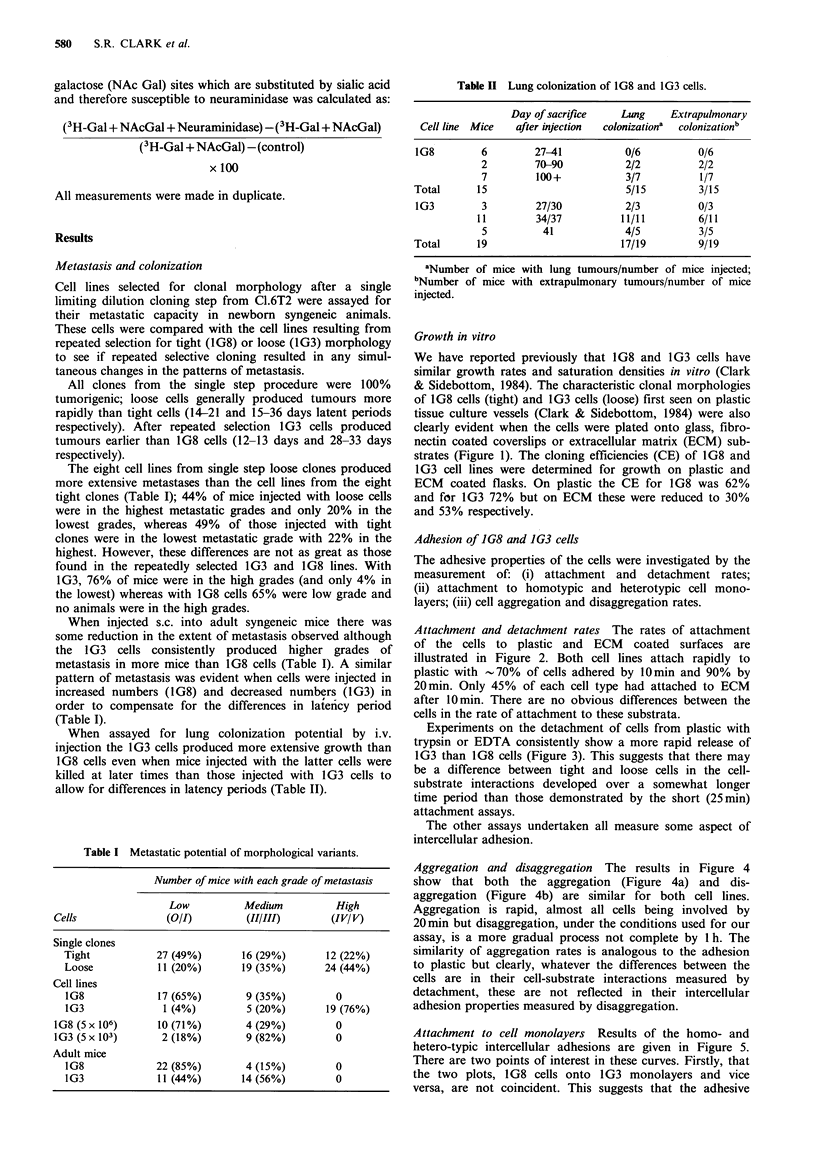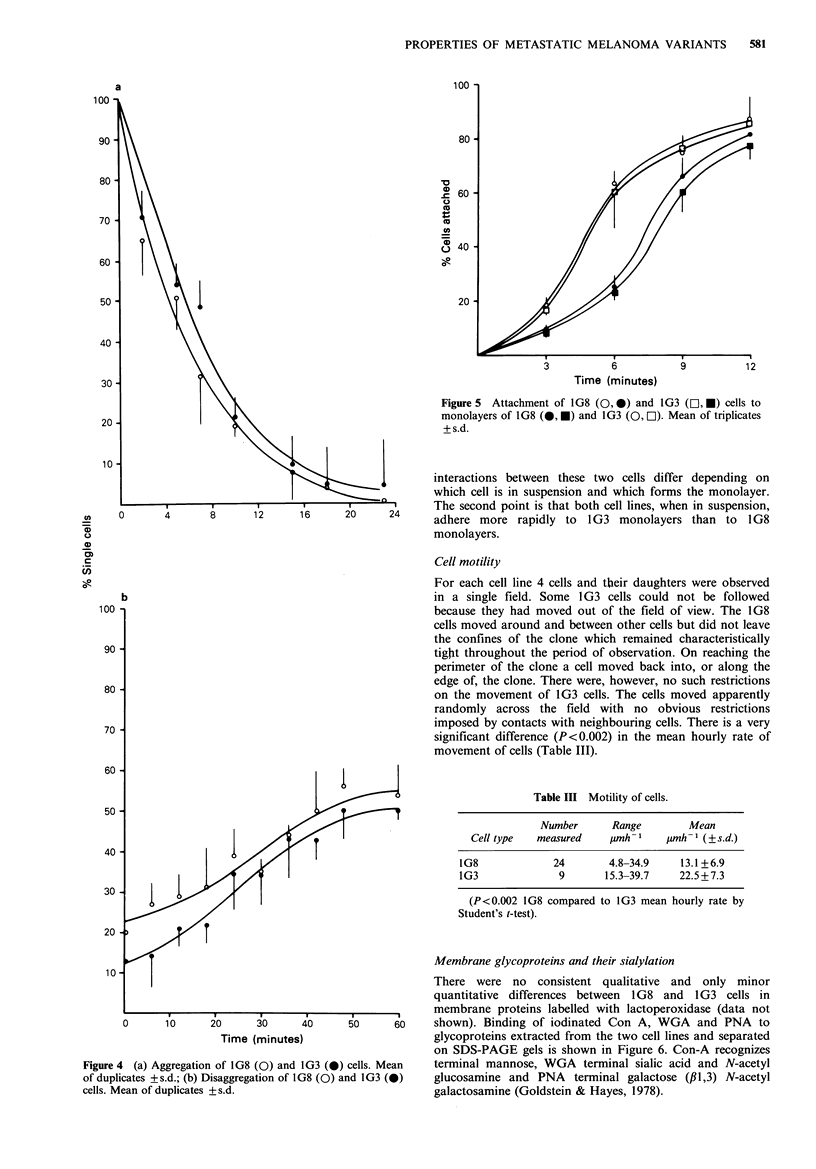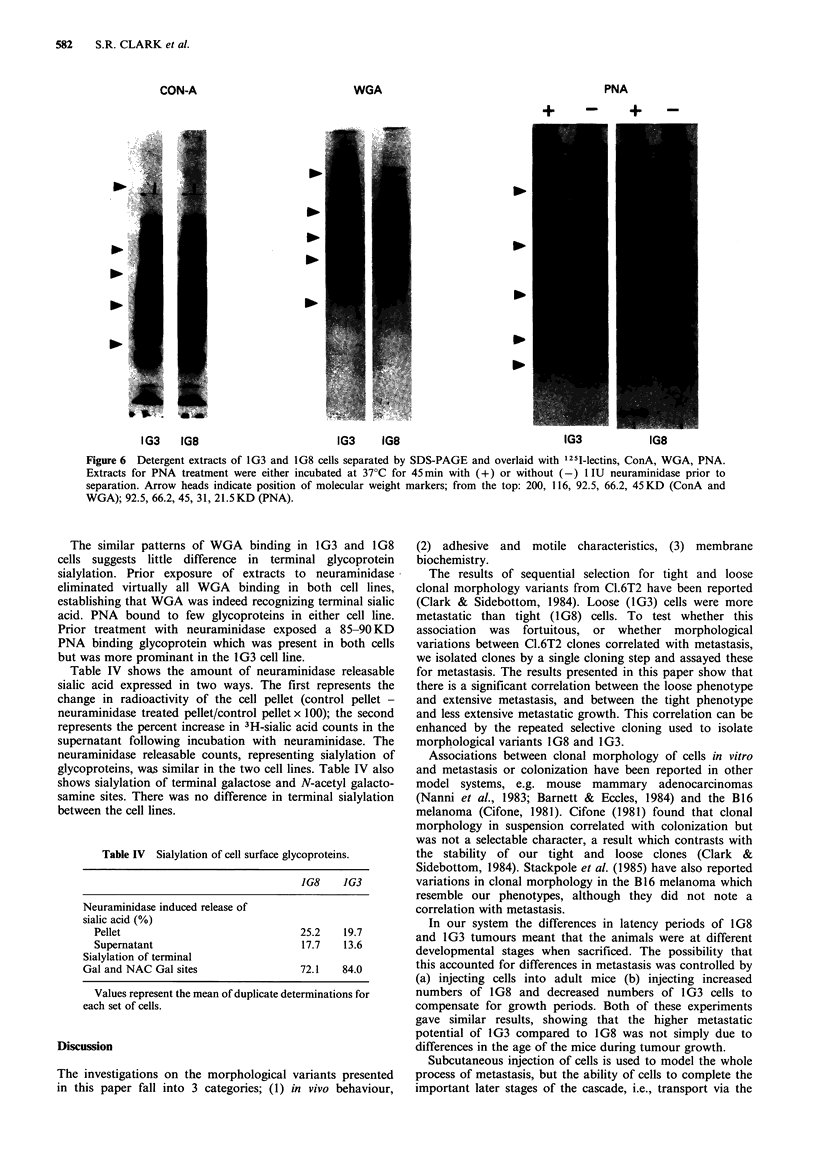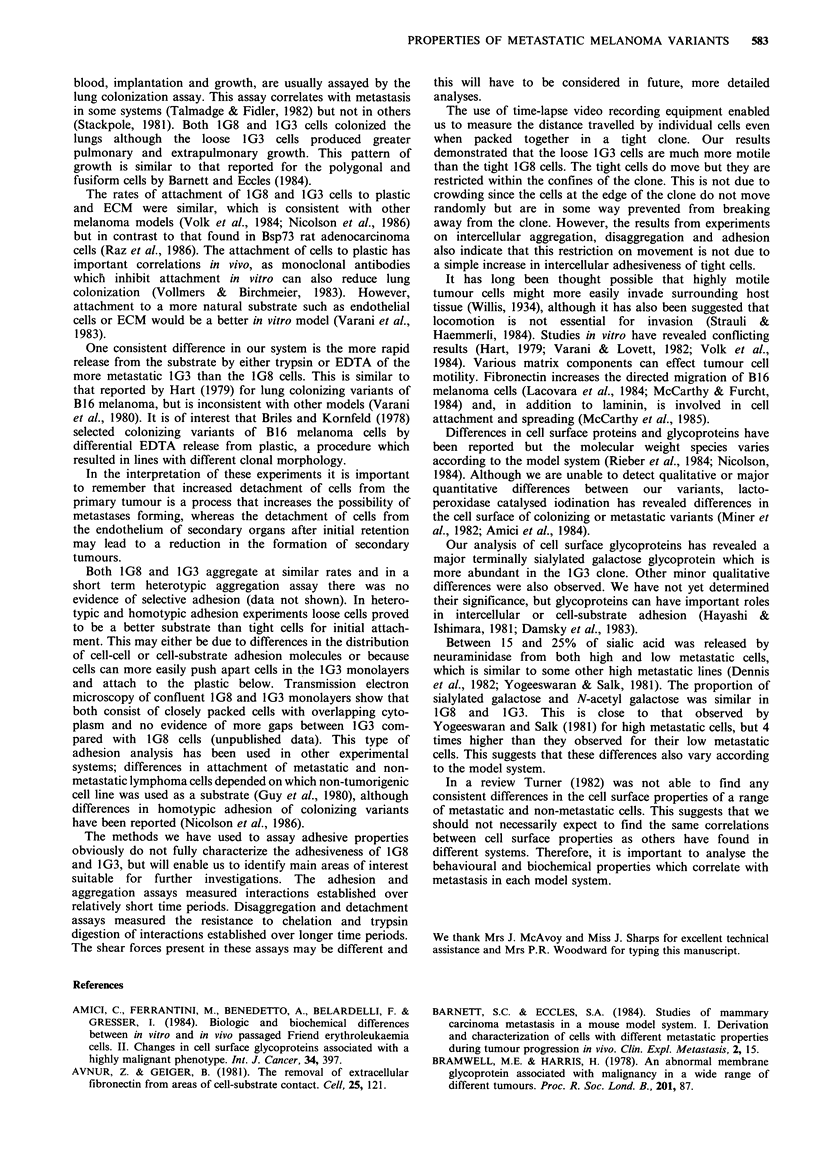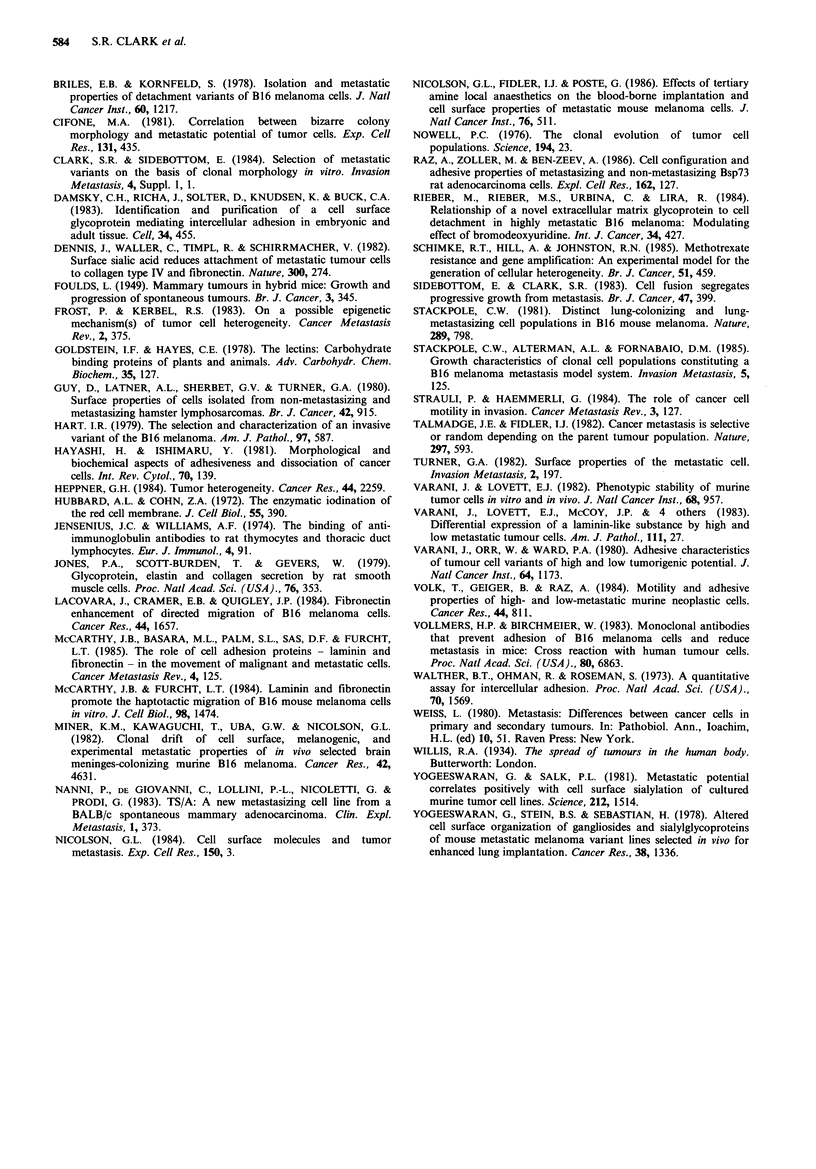# Morphological and metastatic murine melanoma variants: motility, adhesiveness, cell surface and in vivo properties.

**DOI:** 10.1038/bjc.1987.244

**Published:** 1987-11

**Authors:** S. R. Clark, J. S. Brody, E. Sidebottom

**Affiliations:** Sir William Dunn School of Pathology, Oxford, UK.

## Abstract

**Images:**


Br. J. Cancer (1987), 56, 577–584 © The Macmillan Press Ltd., 1987

### Morphological and metastatic murine melanoma variants: Motility, adhesiveness, cell surface and *in vivo* properties

S.R. Clark, J.S. Brody & E. Sidebottom

*Sir William Dunn School of Pathology, South Parks Road, Oxford OX1 3RE, UK.*

**Summary** The behaviour *in vivo* of tight and loose variants of murine melanoma cells is further characterized. *In vitro* clonal morphology is reproduced on a variety of substrates. Results suggest that repeated selection of loose cells can co-select for cells with high metastatic and colonization potentials.

Measurement of cell motility shows that 1G3 (loose) cells are more motile than 1G8 (tight) which are restricted to movements within clonal boundaries.

Studies of adhesive properties show that loose cells are more easily detached from the substrate with trypsin or EDTA and that both cell lines attach more quickly to monolayers of loose cells than to tight ones. No gross differences are found either in attachment rates to plastic and ECM or in aggregation and disaggregation rates.

Analysis of the cell surface has not revealed any differences between 1G8 and 1G3 in the sialylation of terminal galactose and *N*-acetylgalactosamine residues or in neuraminidase releasable sialic acid. The binding patterns of iodinated lectins to SDS-PAGE separated proteins are similar for both lines except for one 85/90 KD protein which is more abundant in 1G3 than 1G8 cells after neuraminidase treatment.

The results show enhanced differences in metastatic potential of tight and loose clones after selective cloning and that there may be important differences in motility and cell-substrate interactions.

Foulds (1949) introduced the term tumour progression to describe the process by which some tumours become more malignant during their growth. Nowell (1976) attributed this to genetic instability, although genetic or epigenetic processes could result in the emergence of variant cells with growth advantages which differ from the main population in the expression of specific characters (Frost & Kerbel, 1983; Schimke *et al.*, 1985).

The existence within tumours of cells heterogeneous for the expression of a wide range of *in vivo* and *in vitro* characters is well documented (for reviews see Heppner, 1984; Nicolson, 1984).

We have previously reported heterogeneity of clonal morphology in a metastatic melanoma × lymphocyte hybrid tumour cell line (Clark & Sidebottom, 1984). These variants grow either as tight or loose colonies and the isolation of cell lines, stable for clonal morphology, which have low or high metastatic phenotypes has been described (Clark & Sidebottom, 1984). The morphology and patterns of growth of these variants suggests a number of ways in which these cells may differ and which might account for their behavioural differences *in vitro*. These include intercellular and cell-substrate adhesion and motility, which in turn are dependent upon cell surface properties. Various stages of the metastatic cascade, for example local invasion or tumour-capillary endothelium adhesion are partially dependent upon intercellular and cell-substrate adhesion and motility (Weiss, 1980; Varani *et al.*, 1980; Nicolson *et al.*, 1986). The cell surface has an important role in these interactions and has been extensively studied for correlations with metastasis and organ colonization (Turner, 1982; Nicolson, 1984).

Observation and measurement of adhesion and motility *in vivo* have inherent difficulties, but these characters can be investigated *in vitro* and some clues about their possible significance *in vivo* thereby gained (Briles & Kornfeld, 1978; Hart, 1979; Vollmers & Birchmeier, 1983; McCarthy *et al.*, 1985).

We have, therefore, looked at the adhesive and motile properties of tight and loose cells and also examined their cell surfaces by lectin binding to PAGE separated glycoproteins, radio-iodination of proteins and quantitation of sialic acid content.

Correspondence: S.R. Clark.
Received 18 December 1986; and in revised form, 12 May 1987.

### Materials and methods

### Cell lines

F87.Cl.6T2 (Cl.6T2) is a metastatic melanoma × lymphocyte hybrid cell line established *in vitro* from a primary tumour (Sidebottom & Clark, 1983). The cell lines 1G8(6F) and 1G3(4E) were selected from F87.Cl.6T2 cells for tight or loose clonal morphology respectively and their *in vitro* and *in vivo* properties have been described elsewhere (Clark & Sidebottom, 1984). Cells were maintained in MEM + 10% newborn calf serum (Flow) (cMEM) and subcultured when confluent; they were regularly tested for mycoplasma and found to be negative. Wiscm cells were derived from explanted hearts from one day old Wistar rats and were maintained in DMEM + 10% foetal calf serum (Gibco) + 10% tryptose phosphate broth (Difco). To produce extracellular matrix (ECM) cells were grown to confluency in 3.5 cm petri dishes and ascorbic acid added for the following 5 days. ECM was prepared according to the method of Jones *et al.* (1979).

### Cloning

Cloning efficiencies were determined by limiting dilution in 96 well microtitre tissue culture plates or by seeding cells into duplicate T75 flasks coated with ECM. Cells were grown for 6 days, fixed, stained with Giemsa and colonies counted.

For single step cloning Cl.6T2 cells were cloned by limiting dilution in microtitre plates. Eight tight and eight loose clones were isolated, grown up and before the fifth passage were assayed for metastasis in newborn mice.

### Fibronectin coated coverslips

Washed 11 mm coverslips (Chance Propper, Warley, UK) were covered with a solution of phosphate buffered saline (PBS pH 7.3) containing 100 $\mu g\,ml^{-1}$ of human plasma fibronectin (Sigma) and incubated for 1 h at room temperature (Avnur & Geiger, 1981). Coverslips were then washed in PBS pH 7.3, placed in 3.5 cm petri dishes containing medium and $10^4$ cells were added and incubated in a 5% $CO_2$ humidified atmosphere.

*Metastasis and lung colonization*

For metastasis assays dissociated cells (>90% viable by trypan blue exclusion test) were injected into the subscapular region of syngeneic (C57Bl x CBA/Ca) mice. Newborn (<5 day) sublethally irradiated (4 Gy) mice received $5 \times 10^4$ and adult (12–16 week) mice $2 \times 10^6$ cells in PBS. Lung colonization was assayed by injecting $5 \times 10^4$ EDTA released cells in 0.1 ml PBS into a lateral tail vein of 12–16 week syngeneic mice.

Metastasis was assessed by examination of the lungs under the dissecting microscope and was confirmed by examination of paraffin embedded haematoxylin and eosin stained sections. A semi-quantitative scale based on the number of metastases seen under the dissecting microscope was used for the assessment of the extent of metastasis: 0 – no lung tumours; I – 1 to 5 tumours; II – 6 to 23; III – 24 to about 50; IV – 50 up to 100; and V – very large numbers (many hundreds). In order to maximize the probability of observing metastases animals were killed when death was impending due to tumour load. Differences in the extent of metastasis were compared using the Kolmogorov–Smirnov two sample test.

*Cell detachment assays*

Cells were grown for 48 h in 3.5 cm 6 well plates to about 80% confluency in cMEM containing 0.1 $\mu$Ci ml$^{-1}$ $^3$H TdR (sp activity = 40 Ci mmol$^{-1}$, Amersham, UK). A single plate was used for each time point with triplicate wells for each cell line. The medium was aspirated, the plates washed with PBS + $Mg^{++}$ and $Ca^{++}$ (PBS-DAB) and 2 ml of either 0.02% EDTA in PBS or 0.125% trypsin in PBS was added to each well and the plates incubated at 37°C on a rotary shaker at 70 r.p.m. At each time point, cells in suspension and those remaining attached were each solubilized with lysis buffer (1% Triton X-100, 1% Na deoxycholate, 0.1% SDS, 0.15 M NaCl, 0.05 M tris HCl (pH 7.2)) and the radioactivity counted in a Packard (Tricarb) scintillation counter. Cell detachment was calculated as

$$\frac{\text{cpm (cells in suspension)}}{\text{total cpm (cells in suspension + cells attached)}} \times 100\%$$

*Cell attachment assays*

Cells were grown in cMEM + 0.1 $\mu$Ci ml$^{-1}$ $^3$H TdR for 48 h and then harvested with 0.02% EDTA/PBS, washed, adjusted to $2.5 \times 10^5$ cells ml$^{-1}$ cMEM and 2 ml was added to each well of a 3.5 cm 6 well tissue culture plate and incubated at 37°C. Each plate consisted of triplicates of each cell line. At various times one plate was removed, swirled gently to resuspend the unattached cells, and the amount of radioactivity in the supernatant and attached fractions was determined. For the homo- and hetero-typic attachment experiments the wells had been previously seeded with cells so that they formed a confluent monolayer at the time of assay (Walther *et al.*, 1973).

*Cell aggregation and disaggregation assays*

Cells harvested with EDTA/PBS were washed, counted, resuspended at $8.5 \times 10^5$ cells ml$^{-1}$ and 2 ml was seeded onto agar in 3.5 cm petri dishes and rotated at 70 r.p.m. at 37°C. At various times the cells were collected, fixed in 10% Formal saline (FS) to prevent further aggregation and the number of single cells counted.

For disaggregation assays cells were grown on 1.5% agar for 48 h. Aggregates were collected, washed, gently resuspended in either 0.02% EDTA/PBS or 0.125% trypsin and were then stirred continuously at 37°C with a rotating magnet. Aliquots of 0.5 ml were taken, fixed and the numbers of single cells counted.

*Cell motility*

Cells were seeded into T25 flasks at $5 \times 10^4$ cells per flask grown for 48 h and then small groups of cells observed on a Leitz diavert microscope. Recording equipment consisted of a National WV1 Newvicon camera, NEC PVC 9507 time lapse recorder and Cotron PM 44B 17″ monitor. The positions of the cells were noted every 3 h on a perspex sheet covering the monitor screen and the distances covered calculated.

*Glycosylation and sialylation of glycoproteins*

Affinity binding of lectins to cellular glycoproteins was carried out as described by Bramwell and Harris (1978). Briefly, $2–5 \times 10^6$ cells were harvested with 0.02% EDTA, washed in PBS and extracted for 20 min at room temperature with 100–200 $\mu$l of 1% Triton X-100 in 10 mM Tris HCl pH 8.0 with 2 mM phenylmethylsulfonyl fluoride. Following centrifugation at 1500 g for 10 min, the supernatant was stored at −20°C until used. The extracts were solubilized in 2% SDS buffer with 0.1 M dithiothreitol, and run on SDS-PAGE gradient slab gels at 40 ma for 2.5 h. The gels were stained with Kenacid Blue, destained, and incubated in PBS + 0.4 M NaCl for 2–3 h. They were then incubated overnight in either ConA, WGA or PNA lectins which had been iodinated to a specific activity of 5–10 $\mu$Ci mg$^{-1}$ by the chloramine-T method (Jensenius & Williams, 1974). Unbound lectin was removed by washing in PBS in 0.4 M NaCl, the gels were dried and exposed to Kodak NS-2T film for 1–4 days. The binding patterns of $^{125}$I labelled lectins shown on the autoradiographs were analyzed with a Joyce–Loebl microdensitometer.

Iodination of cell surface proteins was accomplished using the lactoperoxidase method of Hubbard and Cohn (1972). Cells were harvested with 0.02% EDTA, incubated for 20 min at 4°C in 20 mM glucose with 350 $\mu$g lactoperoxidase, 50 $\mu$g glucose oxidase and 0.5 mCi $^{125}$Iodine (sp. activity $1.35 \times 10^4$ $\mu$Ci mg$^{-1}$), and then extracted as above with Triton X-100. Gradient gels were run and autoradiographs prepared as above.

Cell surface sialylation was evaluated by two methods. The first measured neuraminidase-induced release of labelled sialic acid from cells (Dennis *et al.*, 1982). Fresh medium containing 2 $\mu$Ci ml$^{-1}$ [6-$^3$H]N-acetylmannosamine (sp. activity 228 Ci mM$^{-1}$) was added to subconfluent cells grown in petri dishes. After 48 h the cells were washed, harvested with 0.02% EDTA, washed in PBS and $1.2 \times 10^6$ cells were placed in 100 $\mu$l PBS with $Ca^{++}$ and $Mg^{++}$ at pH 6.1. To half the cells 0.02 IU neuraminidase (Calbiochem) was added and the cells were incubated on a shaker at 37°C for 60 min. They were then washed twice in PBS with $Ca^{++}$ and $Mg^{++}$ pH 7.3. The supernatant was saved, the pellet solubilized and radioactivity in both fractions was measured. The percentage of neuraminidase accessible sialic acid was calculated as:

$$\frac{\text{control pellet-neuraminidase pellet}}{\text{control pellet}} \times 100\text{; or}$$

% of total counts in neuraminidase supernatant – % of total counts in control supernatant. All measurements were made in duplicate.

The second method used measured sialylation of galactose and *N*-acetyl galactose sites (Yogeeswaran *et al.*, 1978). Cells were harvested with 0.02% EDTA and $6–8 \times 10^6$ cells were incubated for 1 h at 37°C on a shaker in PBS with $Ca^{++}$ and $Mg^{++}$, pH 6.1 with 5 units galactose oxidase in the presence or absence of 0.03 IU neuraminidase. Control cells contained neither galactose oxidase nor neuraminidase. After cells were washed they were incubated for 30 min at 37°C with 1.5 mCi sodium boro[$^3$H]hydride (sp. activity 20 Ci mM$^{-1}$), washed three times, solubilized and radioactivity measured. The percent of $^3$H labelled galactose (Gal) and *N*-acetyl

**Figure 1** Clonal morphology of 1G8 cells (a, b, c) and 1G3 cells (d, e, f) on glass (a, d), fibronectin coated coverslips (b, e) and Wiscm matrix (c, f). Phase contrast, Bar = 100 μm.

**Figure 2** Attachment of 1G8 (○, □) and 1G3 (●, ■) to plastic (○, ●) and Wiscm matrix (□, ■) substrates. Mean of duplicates ±s.d.

**Figure 3** Detachment of 1G8 (○, □) and 1G3 (●, ■) from plastic by trypsin (□, ■) or EDTA (○, ●). Mean of triplicates ±s.d.

galactose (NAc Gal) sites which are substituted by sialic acid and therefore susceptible to neuraminidase was calculated as:

$$\frac{(^3\text{H-Gal} + \text{NAcGal} + \text{Neuraminidase}) - (^3\text{H-Gal} + \text{NAcGal})}{(^3\text{H-Gal} + \text{NAcGal}) - (\text{control})} \times 100$$

All measurements were made in duplicate.

### Results

### *Metastasis and colonization*

Cell lines selected for clonal morphology after a single limiting dilution cloning step from Cl.6T2 were assayed for their metastatic capacity in newborn syngeneic animals. These cells were compared with the cell lines resulting from repeated selection for tight (1G8) or loose (1G3) morphology to see if repeated selective cloning resulted in any simultaneous changes in the patterns of metastasis.

All clones from the single step procedure were 100% tumorigenic; loose cells generally produced tumours more rapidly than tight cells (14–21 and 15–36 days latent periods respectively). After repeated selection 1G3 cells produced tumours earlier than 1G8 cells (12–13 days and 28–33 days respectively).

The eight cell lines from single step loose clones produced more extensive metastases than the cell lines from the eight tight clones (Table I); 44% of mice injected with loose cells were in the highest metastatic grades and only 20% in the lowest grades, whereas 49% of those injected with tight clones were in the lowest metastatic grade with 22% in the highest. However, these differences are not as great as those found in the repeatedly selected 1G3 and 1G8 lines. With 1G3, 76% of mice were in the high grades (and only 4% in the lowest) whereas with 1G8 cells 65% were low grade and no animals were in the high grades.

When injected s.c. into adult syngeneic mice there was some reduction in the extent of metastasis observed although the 1G3 cells consistently produced higher grades of metastasis in more mice than 1G8 cells (Table I). A similar pattern of metastasis was evident when cells were injected in increased numbers (1G8) and decreased numbers (1G3) in order to compensate for the differences in latency period (Table I).

When assayed for lung colonization potential by i.v. injection the 1G3 cells produced more extensive growth than 1G8 cells even when mice injected with the latter cells were killed at later times than those injected with 1G3 cells to allow for differences in latency periods (Table II).

**Table I** Metastatic potential of morphological variants.

| Cells | Number of mice with each grade of metastasis: Low (O/I) | Medium (II/III) | High (IV/V) |
|---|---|---|---|
| Single clones | | | |
| Tight | 27 (49%) | 16 (29%) | 12 (22%) |
| Loose | 11 (20%) | 19 (35%) | 24 (44%) |
| Cell lines | | | |
| 1G8 | 17 (65%) | 9 (35%) | 0 |
| 1G3 | 1 (4%) | 5 (20%) | 19 (76%) |
| 1G8 ($5\times10^6$) | 10 (71%) | 4 (29%) | 0 |
| 1G3 ($5\times10^3$) | 2 (18%) | 9 (82%) | 0 |
| Adult mice | | | |
| 1G8 | 22 (85%) | 4 (15%) | 0 |
| 1G3 | 11 (44%) | 14 (56%) | 0 |

**Table II** Lung colonization of 1G8 and 1G3 cells.

| Cell line | Mice | Day of sacrifice after injection | Lung colonization[a] | Extrapulmonary colonization[b] |
|---|---|---|---|---|
| 1G8 | 6 | 27–41 | 0/6 | 0/6 |
| | 2 | 70–90 | 2/2 | 2/2 |
| | 7 | 100+ | 3/7 | 1/7 |
| Total | 15 | | 5/15 | 3/15 |
| 1G3 | 3 | 27/30 | 2/3 | 0/3 |
| | 11 | 34/37 | 11/11 | 6/11 |
| | 5 | 41 | 4/5 | 3/5 |
| Total | 19 | | 17/19 | 9/19 |

[a]Number of mice with lung tumours/number of mice injected; [b]Number of mice with extrapulmonary tumours/number of mice injected.

### *Growth in vitro*

We have reported previously that 1G8 and 1G3 cells have similar growth rates and saturation densities *in vitro* (Clark & Sidebottom, 1984). The characteristic clonal morphologies of 1G8 cells (tight) and 1G3 cells (loose) first seen on plastic tissue culture vessels (Clark & Sidebottom, 1984) were also clearly evident when the cells were plated onto glass, fibronectin coated coverslips or extracellular matrix (ECM) substrates (Figure 1). The cloning efficiencies (CE) of 1G8 and 1G3 cell lines were determined for growth on plastic and ECM coated flasks. On plastic the CE for 1G8 was 62% and for 1G3 72% but on ECM these were reduced to 30% and 53% respectively.

### *Adhesion of 1G8 and 1G3 cells*

The adhesive properties of the cells were investigated by the measurement of: (i) attachment and detachment rates; (ii) attachment to homotypic and heterotypic cell monolayers; (iii) cell aggregation and disaggregation rates.

*Attachment and detachment rates* The rates of attachment of the cells to plastic and ECM coated surfaces are illustrated in Figure 2. Both cell lines attach rapidly to plastic with ~70% of cells adhered by 10 min and 90% by 20 min. Only 45% of each cell type had attached to ECM after 10 min. There are no obvious differences between the cells in the rate of attachment to these substrata.

Experiments on the detachment of cells from plastic with trypsin or EDTA consistently show a more rapid release of 1G3 than 1G8 cells (Figure 3). This suggests that there may be a difference between tight and loose cells in the cell-substrate interactions developed over a somewhat longer time period than those demonstrated by the short (25 min) attachment assays.

The other assays undertaken all measure some aspect of intercellular adhesion.

*Aggregation and disaggregation* The results in Figure 4 show that both the aggregation (Figure 4a) and disaggregation (Figure 4b) are similar for both cell lines. Aggregation is rapid, almost all cells being involved by 20 min but disaggregation, under the conditions used for our assay, is a more gradual process not complete by 1 h. The similarity of aggregation rates is analogous to the adhesion to plastic but clearly, whatever the differences between the cells are in their cell-substrate interactions measured by detachment, these are not reflected in their intercellular adhesion properties measured by disaggregation.

*Attachment to cell monolayers* Results of the homo- and hetero-typic intercellular adhesions are given in Figure 5. There are two points of interest in these curves. Firstly, that the two plots, 1G8 cells onto 1G3 monolayers and vice versa, are not coincident. This suggests that the adhesive

Figure 4 (a) Aggregation of 1G8 (○) and 1G3 (●) cells. Mean of duplicates ±s.d.; (b) Disaggregation of 1G8 (○) and 1G3 (●) cells. Mean of duplicates ±s.d.

**Figure 5** Attachment of 1G8 (○, ●) and 1G3 (□, ■) cells to monolayers of 1G8 (●, ■) and 1G3 (○, □). Mean of triplicates ±s.d.

interactions between these two cells differ depending on which cell is in suspension and which forms the monolayer. The second point is that both cell lines, when in suspension, adhere more rapidly to 1G3 monolayers than to 1G8 monolayers.

*Cell motility*

For each cell line 4 cells and their daughters were observed in a single field. Some 1G3 cells could not be followed because they had moved out of the field of view. The 1G8 cells moved around and between other cells but did not leave the confines of the clone which remained characteristically tight throughout the period of observation. On reaching the perimeter of the clone a cell moved back into, or along the edge of, the clone. There were, however, no such restrictions on the movement of 1G3 cells. The cells moved apparently randomly across the field with no obvious restrictions imposed by contacts with neighbouring cells. There is a very significant difference ($P<0.002$) in the mean hourly rate of movement of cells (Table III).

**Table III** Motility of cells.

| *Cell type* | *Number measured* | *Range* $\mu mh^{-1}$ | *Mean* $\mu mh^{-1}$ ($\pm s.d.$) |
|---|---|---|---|
| 1G8 | 24 | 4.8–34.9 | 13.1±6.9 |
| 1G3 | 9 | 15.3–39.7 | 22.5±7.3 |

($P<0.002$ 1G8 compared to 1G3 mean hourly rate by Student's *t*-test).

*Membrane glycoproteins and their sialylation*

There were no consistent qualitative and only minor quantitative differences between 1G8 and 1G3 cells in membrane proteins labelled with lactoperoxidase (data not shown). Binding of iodinated Con A, WGA and PNA to glycoproteins extracted from the two cell lines and separated on SDS-PAGE gels is shown in Figure 6. Con-A recognizes terminal mannose, WGA terminal sialic acid and *N*-acetyl glucosamine and PNA terminal galactose ($\beta$1,3) *N*-acetyl galactosamine (Goldstein & Hayes, 1978).

**Figure 6** Detergent extracts of 1G3 and 1G8 cells separated by SDS-PAGE and overlaid with $^{125}$I-lectins, ConA, WGA, PNA. Extracts for PNA treatment were either incubated at 37°C for 45min with (+) or without (−) 1IU neuraminidase prior to separation. Arrow heads indicate position of molecular weight markers; from the top: 200, 116, 92.5, 66.2, 45KD (ConA and WGA); 92.5, 66.2, 45, 31, 21.5KD (PNA).

The similar patterns of WGA binding in 1G3 and 1G8 cells suggests little difference in terminal glycoprotein sialylation. Prior exposure of extracts to neuraminidase eliminated virtually all WGA binding in both cell lines, establishing that WGA was indeed recognizing terminal sialic acid. PNA bound to few glycoproteins in either cell line. Prior treatment with neuraminidase exposed a 85–90KD PNA binding glycoprotein which was present in both cells but was more prominant in the 1G3 cell line.

Table IV shows the amount of neuraminidase releasable sialic acid expressed in two ways. The first represents the change in radioactivity of the cell pellet (control pellet − neuraminidase treated pellet/control pellet × 100); the second represents the percent increase in $^{3}$H-sialic acid counts in the supernatant following incubation with neuraminidase. The neuraminidase releasable counts, representing sialylation of glycoproteins, was similar in the two cell lines. Table IV also shows sialylation of terminal galactose and *N*-acetyl galactosamine sites. There was no difference in terminal sialylation between the cell lines.

**Table IV** Sialylation of cell surface glycoproteins.

| | *1G8* | *1G3* |
|---|---|---|
| Neuraminidase induced release of sialic acid (%) | | |
| Pellet | 25.2 | 19.7 |
| Supernatant | 17.7 | 13.6 |
| Sialylation of terminal Gal and NAC Gal sites | 72.1 | 84.0 |

Values represent the mean of duplicate determinations for each set of cells.

### Discussion

The investigations on the morphological variants presented in this paper fall into 3 categories; (1) *in vivo* behaviour, (2) adhesive and motile characteristics, (3) membrane biochemistry.

The results of sequential selection for tight and loose clonal morphology variants from Cl.6T2 have been reported (Clark & Sidebottom, 1984). Loose (1G3) cells were more metastatic than tight (1G8) cells. To test whether this association was fortuitous, or whether morphological variations between Cl.6T2 clones correlated with metastasis, we isolated clones by a single cloning step and assayed these for metastasis. The results presented in this paper show that there is a significant correlation between the loose phenotype and extensive metastasis, and between the tight phenotype and less extensive metastatic growth. This correlation can be enhanced by the repeated selective cloning used to isolate morphological variants 1G8 and 1G3.

Associations between clonal morphology of cells *in vitro* and metastasis or colonization have been reported in other model systems, e.g. mouse mammary adenocarcinomas (Nanni *et al.*, 1983; Barnett & Eccles, 1984) and the B16 melanoma (Cifone, 1981). Cifone (1981) found that clonal morphology in suspension correlated with colonization but was not a selectable character, a result which contrasts with the stability of our tight and loose clones (Clark & Sidebottom, 1984). Stackpole *et al.* (1985) have also reported variations in clonal morphology in the B16 melanoma which resemble our phenotypes, although they did not note a correlation with metastasis.

In our system the differences in latency periods of 1G8 and 1G3 tumours meant that the animals were at different developmental stages when sacrificed. The possibility that this accounted for differences in metastasis was controlled by (a) injecting cells into adult mice (b) injecting increased numbers of 1G8 and decreased numbers of 1G3 cells to compensate for growth periods. Both of these experiments gave similar results, showing that the higher metastatic potential of 1G3 compared to 1G8 was not simply due to differences in the age of the mice during tumour growth.

Subcutaneous injection of cells is used to model the whole process of metastasis, but the ability of cells to complete the important later stages of the cascade, i.e., transport via the blood, implantation and growth, are usually assayed by the lung colonization assay. This assay correlates with metastasis in some systems (Talmadge & Fidler, 1982) but not in others (Stackpole, 1981). Both 1G8 and 1G3 cells colonized the lungs although the loose 1G3 cells produced greater pulmonary and extrapulmonary growth. This pattern of growth is similar to that reported for the polygonal and fusiform cells by Barnett and Eccles (1984).

The rates of attachment of 1G8 and 1G3 cells to plastic and ECM were similar, which is consistent with other melanoma models (Volk *et al.*, 1984; Nicolson *et al.*, 1986) but in contrast to that found in Bsp73 rat adenocarcinoma cells (Raz *et al.*, 1986). The attachment of cells to plastic has important correlations *in vivo*, as monoclonal antibodies which inhibit attachment *in vitro* can also reduce lung colonization (Vollmers & Birchmeier, 1983). However, attachment to a more natural substrate such as endothelial cells or ECM would be a better *in vitro* model (Varani *et al.*, 1983).

One consistent difference in our system is the more rapid release from the substrate by either trypsin or EDTA of the more metastatic 1G3 than the 1G8 cells. This is similar to that reported by Hart (1979) for lung colonizing variants of B16 melanoma, but is inconsistent with other models (Varani *et al.*, 1980). It is of interest that Briles and Kornfeld (1978) selected colonizing variants of B16 melanoma cells by differential EDTA release from plastic, a procedure which resulted in lines with different clonal morphology.

In the interpretation of these experiments it is important to remember that increased detachment of cells from the primary tumour is a process that increases the possibility of metastases forming, whereas the detachment of cells from the endothelium of secondary organs after initial retention may lead to a reduction in the formation of secondary tumours.

Both 1G8 and 1G3 aggregate at similar rates and in a short term heterotypic aggregation assay there was no evidence of selective adhesion (data not shown). In heterotypic and homotypic adhesion experiments loose cells proved to be a better substrate than tight cells for initial attachment. This may either be due to differences in the distribution of cell-cell or cell-substrate adhesion molecules or because cells can more easily push apart cells in the 1G3 monolayers and attach to the plastic below. Transmission electron microscopy of confluent 1G8 and 1G3 monolayers show that both consist of closely packed cells with overlapping cytoplasm and no evidence of more gaps between 1G3 compared with 1G8 cells (unpublished data). This type of adhesion analysis has been used in other experimental systems; differences in attachment of metastatic and non-metastatic lymphoma cells depended on which non-tumorigenic cell line was used as a substrate (Guy *et al.*, 1980), although differences in homotypic adhesion of colonizing variants have been reported (Nicolson *et al.*, 1986).

The methods we have used to assay adhesive properties obviously do not fully characterize the adhesiveness of 1G8 and 1G3, but will enable us to identify main areas of interest suitable for further investigations. The adhesion and aggregation assays measured interactions established over relatively short time periods. Disaggregation and detachment assays measured the resistance to chelation and trypsin digestion of interactions established over longer time periods. The shear forces present in these assays may be different and this will have to be considered in future, more detailed analyses.

The use of time-lapse video recording equipment enabled us to measure the distance travelled by individual cells even when packed together in a tight clone. Our results demonstrated that the loose 1G3 cells are much more motile than the tight 1G8 cells. The tight cells do move but they are restricted within the confines of the clone. This is not due to crowding since the cells at the edge of the clone do not move randomly but are in some way prevented from breaking away from the clone. However, the results from experiments on intercellular aggregation, disaggregation and adhesion also indicate that this restriction on movement is not due to a simple increase in intercellular adhesiveness of tight cells.

It has long been thought possible that highly motile tumour cells might more easily invade surrounding host tissue (Willis, 1934), although it has also been suggested that locomotion is not essential for invasion (Strauli & Haemmerli, 1984). Studies *in vitro* have revealed conflicting results (Hart, 1979; Varani & Lovett, 1982; Volk *et al.*, 1984). Various matrix components can effect tumour cell motility. Fibronectin increases the directed migration of B16 melanoma cells (Lacovara *et al.*, 1984; McCarthy & Furcht, 1984) and, in addition to laminin, is involved in cell attachment and spreading (McCarthy *et al.*, 1985).

Differences in cell surface proteins and glycoproteins have been reported but the molecular weight species varies according to the model system (Rieber *et al.*, 1984; Nicolson, 1984). Although we are unable to detect qualitative or major quantitative differences between our variants, lacto-peroxidase catalysed iodination has revealed differences in the cell surface of colonizing or metastatic variants (Miner *et al.*, 1982; Amici *et al.*, 1984).

Our analysis of cell surface glycoproteins has revealed a major terminally sialylated galactose glycoprotein which is more abundant in the 1G3 clone. Other minor qualitative differences were also observed. We have not yet determined their significance, but glycoproteins can have important roles in intercellular or cell-substrate adhesion (Hayashi & Ishimara, 1981; Damsky *et al.*, 1983).

Between 15 and 25% of sialic acid was released by neuraminidase from both high and low metastatic cells, which is similar to some other high metastatic lines (Dennis *et al.*, 1982; Yogeeswaran & Salk, 1981). The proportion of sialylated galactose and *N*-acetyl galactose was similar in 1G8 and 1G3. This is close to that observed by Yogeeswaran and Salk (1981) for high metastatic cells, but 4 times higher than they observed for their low metastatic cells. This suggests that these differences also vary according to the model system.

In a review Turner (1982) was not able to find any consistent differences in the cell surface properties of a range of metastatic and non-metastatic cells. This suggests that we should not necessarily expect to find the same correlations between cell surface properties as others have found in different systems. Therefore, it is important to analyse the behavioural and biochemical properties which correlate with metastasis in each model system.

We thank Mrs J. McAvoy and Miss J. Sharps for excellent technical assistance and Mrs P.R. Woodward for typing this manuscript.

### References

AMICI, C., FERRANTINI, M., BENEDETTO, A., BELARDELLI, F. & GRESSER, I. (1984). Biologic and biochemical differences between *in vitro* and *in vivo* passaged Friend erythroleukaemia cells. II. Changes in cell surface glycoproteins associated with a highly malignant phenotype. *Int. J. Cancer*, **34**, 397.

AVNUR, Z. & GEIGER, B. (1981). The removal of extracellular fibronectin from areas of cell-substrate contact. *Cell*, **25**, 121.

BARNETT, S.C. & ECCLES, S.A. (1984). Studies of mammary carcinoma metastasis in a mouse model system. I. Derivation and characterization of cells with different metastatic properties during tumour progression *in vivo*. *Clin. Expl. Metastasis*, **2**, 15.

BRAMWELL, M.E. & HARRIS, H. (1978). An abnormal membrane glycoprotein associated with malignancy in a wide range of different tumours. *Proc. R. Soc. Lond. B.*, **201**, 87.

BRILES, E.B. & KORNFELD, S. (1978). Isolation and metastatic properties of detachment variants of B16 melanoma cells. *J. Natl Cancer Inst.*, **60**, 1217.

CIFONE, M.A. (1981). Correlation between bizarre colony morphology and metastatic potential of tumor cells. *Exp. Cell Res.*, **131**, 435.

CLARK, S.R. & SIDEBOTTOM, E. (1984). Selection of metastatic variants on the basis of clonal morphology *in vitro*. *Invasion Metastasis*, **4**, Suppl. 1, 1.

DAMSKY, C.H., RICHA, J., SOLTER, D., KNUDSEN, K. & BUCK, C.A. (1983). Identification and purification of a cell surface glycoprotein mediating intercellular adhesion in embryonic and adult tissue. *Cell*, **34**, 455.

DENNIS, J., WALLER, C., TIMPL, R. & SCHIRRMACHER, V. (1982). Surface sialic acid reduces attachment of metastatic tumour cells to collagen type IV and fibronectin. *Nature*, **300**, 274.

FOULDS, L. (1949). Mammary tumours in hybrid mice: Growth and progression of spontaneous tumours. *Br. J. Cancer*, **3**, 345.

FROST, P. & KERBEL, R.S. (1983). On a possible epigenetic mechanism(s) of tumor cell heterogeneity. *Cancer Metastasis Rev.*, **2**, 375.

GOLDSTEIN, I.F. & HAYES, C.E. (1978). The lectins: Carbohydrate binding proteins of plants and animals. *Adv. Carbohydr. Chem. Biochem.*, **35**, 127.

GUY, D., LATNER, A.L., SHERBET, G.V. & TURNER, G.A. (1980). Surface properties of cells isolated from non-metastasizing and metastasizing hamster lymphosarcomas. *Br. J. Cancer*, **42**, 915.

HART. I.R. (1979). The selection and characterization of an invasive variant of the B16 melanoma. *Am. J. Pathol.*, **97**, 587.

HAYASHI, H. & ISHIMARU, Y. (1981). Morphological and biochemical aspects of adhesiveness and dissociation of cancer cells. *Int. Rev. Cytol.*, **70**, 139.

HEPPNER, G.H. (1984). Tumor heterogeneity. *Cancer Res.*, **44**, 2259.

HUBBARD, A.L. & COHN, Z.A. (1972). The enzymatic iodination of the red cell membrane. *J. Cell Biol.*, **55**, 390.

JENSENIUS, J.C. & WILLIAMS, A.F. (1974). The binding of anti-immunoglobulin antibodies to rat thymocytes and thoracic duct lymphocytes. *Eur. J. Immunol.*, **4**, 91.

JONES, P.A., SCOTT-BURDEN, T. & GEVERS, W. (1979). Glycoprotein, elastin and collagen secretion by rat smooth muscle cells. *Proc. Natl Acad. Sci. (USA).*, **76**, 353.

LACOVARA, J., CRAMER, E.B. & QUIGLEY, J.P. (1984). Fibronectin enhancement of directed migration of B16 melanoma cells. *Cancer Res.*, **44**, 1657.

McCARTHY, J.B., BASARA, M.L., PALM, S.L., SAS, D.F. & FURCHT, L.T. (1985). The role of cell adhesion proteins – laminin and fibronectin – in the movement of malignant and metastatic cells. *Cancer Metastasis Rev.*, **4**, 125.

McCARTHY, J.B. & FURCHT, L.T. (1984). Laminin and fibronectin promote the haptotactic migration of B16 mouse melanoma cells *in vitro*. *J. Cell Biol.*, **98**, 1474.

MINER, K.M., KAWAGUCHI, T., UBA, G.W. & NICOLSON, G.L. (1982). Clonal drift of cell surface, melanogenic, and experimental metastatic properties of *in vivo* selected brain meninges-colonizing murine B16 melanoma. *Cancer Res.*, **42**, 4631.

NANNI, P., DE GIOVANNI, C., LOLLINI, P.-L., NICOLETTI, G. & PRODI, G. (1983). TS/A: A new metastasizing cell line from a BALB/c spontaneous mammary adenocarcinoma. *Clin. Expl. Metastasis*, **1**, 373.

NICOLSON, G.L. (1984). Cell surface molecules and tumor metastasis. *Exp. Cell Res.*, **150**, 3.

NICOLSON, G.L., FIDLER, I.J. & POSTE, G. (1986). Effects of tertiary amine local anaesthetics on the blood-borne implantation and cell surface properties of metastatic mouse melanoma cells. *J. Natl Cancer Inst.*, **76**, 511.

NOWELL, P.C. (1976). The clonal evolution of tumor cell populations. *Science*, **194**, 23.

RAZ, A., ZOLLER, M. & BEN-ZEEV, A. (1986). Cell configuration and adhesive properties of metastasizing and non-metastasizing Bsp73 rat adenocarcinoma cells. *Expl. Cell Res.*, **162**, 127.

RIEBER, M., RIEBER, M.S., URBINA, C. & LIRA, R. (1984). Relationship of a novel extracellular matrix glycoprotein to cell detachment in highly metastatic B16 melanoma: Modulating effect of bromodeoxyuridine. *Int. J. Cancer*, **34**, 427.

SCHIMKE, R.T., HILL, A. & JOHNSTON, R.N. (1985). Methotrexate resistance and gene amplification: An experimental model for the generation of cellular heterogeneity. *Br. J. Cancer*, **51**, 459.

SIDEBOTTOM, E. & CLARK, S.R. (1983). Cell fusion segregates progressive growth from metastasis. *Br. J. Cancer*, **47**, 399.

STACKPOLE, C.W. (1981). Distinct lung-colonizing and lung-metastasizing cell populations in B16 mouse melanoma. *Nature*, **289**, 798.

STACKPOLE, C.W., ALTERMAN, A.L. & FORNABAIO, D.M. (1985). Growth characteristics of clonal cell populations constituting a B16 melanoma metastasis model system. *Invasion Metastasis*, **5**, 125.

STRAULI, P. & HAEMMERLI, G. (1984). The role of cancer cell motility in invasion. *Cancer Metastasis Rev.*, **3**, 127.

TALMADGE, J.E. & FIDLER, I.J. (1982). Cancer metastasis is selective or random depending on the parent tumour population. *Nature*, **297**, 593.

TURNER, G.A. (1982). Surface properties of the metastatic cell. *Invasion Metastasis*, **2**, 197.

VARANI, J. & LOVETT, E.J. (1982). Phenotypic stability of murine tumor cells *in vitro* and *in vivo*. *J. Natl Cancer Inst.*, **68**, 957.

VARANI, J., LOVETT, E.J., McCOY, J.P. & 4 others (1983). Differential expression of a laminin-like substance by high and low metastatic tumour cells. *Am. J. Pathol.*, **111**, 27.

VARANI, J., ORR, W. & WARD, P.A. (1980). Adhesive characteristics of tumour cell variants of high and low tumorigenic potential. *J. Natl Cancer Inst.*, **64**, 1173.

VOLK, T., GEIGER, B. & RAZ, A. (1984). Motility and adhesive properties of high- and low-metastatic murine neoplastic cells. *Cancer Res.*, **44**, 811.

VOLLMERS, H.P. & BIRCHMEIER, W. (1983). Monoclonal antibodies that prevent adhesion of B16 melanoma cells and reduce metastasis in mice: Cross reaction with human tumour cells. *Proc. Natl Acad. Sci. (USA).*, **80**, 6863.

WALTHER, B.T., OHMAN, R. & ROSEMAN, S. (1973). A quantitative assay for intercellular adhesion. *Proc. Natl Acad. Sci. (USA).*, **70**, 1569.

WEISS, L. (1980). Metastasis: Differences between cancer cells in primary and secondary tumours. In: Pathobiol. Ann., Ioachim, H.L. (ed) **10**, 51. Raven Press: New York.

WILLIS, R.A. (1934). *The spread of tumours in the human body*. Butterworth: London.

YOGEESWARAN, G. & SALK, P.L. (1981). Metastatic potential correlates positively with cell surface sialylation of cultured murine tumor cell lines. *Science*, **212**, 1514.

YOGEESWARAN, G., STEIN, B.S. & SEBASTIAN, H. (1978). Altered cell surface organization of gangliosides and sialylglycoproteins of mouse metastatic melanoma variant lines selected *in vivo* for enhanced lung implantation. *Cancer Res.*, **38**, 1336.